# A pilot study investigating the comparison of immunological responses to two immunosuppressive regimens in a porcine model

**DOI:** 10.3389/fvets.2026.1816404

**Published:** 2026-06-17

**Authors:** María Pulido, Claudia Báez-Díaz, Verónica Álvarez, Fátima Vázquez-López, Joaquim Vives, Angel Raya, Javier G Casado, Francisco Miguel Sánchez-Margallo, Verónica Crisóstomo, Esther López

**Affiliations:** 1 Jesus Usón Minimally Invasive Surgery Centre, Cáceres, Spain; 2 Institute of Molecular Pathology Biomarkers, Universidad de Extremadura, Cáceres, Spain; 3 Banc de Sang i Teixits (BST), Musculoskeletal Tissue Engineering Group, Vall d'Hebron Research Institute (VHIR), Departament de Medicina, Universitat Autònoma de Barcelona, Barcelona, Spain; 4 Regenerative Medicine Program and Program for Clinical Translation of Regenerative Medicine in Catalonia-P-CMR[C], Bellvitge Institute for Biomedical Research (IDIBELL), Catalan Institution for Research and Advanced Studies (ICREA), Barcelona, Spain; 5 Biomedical Research Network Centre in Bioengineering, Nanomaterials, and Nanomedicine (CIBER-BBN), Instituto de Salud Carlos III, Madrid, Spain; 6 Immunology Unit, Department of Physiology, Universidad de Extremadura, Cáceres, Spain

**Keywords:** cell therapy, immune subsets, immunosuppression, lymphopenia, porcine model

## Abstract

Effective immunosuppression in large animal models is essential for successful preclinical assessment of cell-based therapies in the allo- or xeno-transplantation setting. Pigs, due to their physiological and immunological similarity to humans, are widely used in translational research. However, direct, longitudinal comparisons of immunosuppressive strategies in swine are scarce, limiting the development of safe and reliable protocols. To address this gap, we performed a head-to-head comparison of two immunosuppressive regimens in pigs (*n* = 8; 4 animals per group) to identify a protocol that achieves robust immune modulation with minimal systemic toxicity. Protocol I consisted of an intravenous induction with mycophenolate mofetil (MMF) and methylprednisolone, followed by oral MMF and tacrolimus. Protocol II included intravenous abatacept and methylprednisolone, two booster doses of abatacept, and daily oral cyclosporine A. Both regimens were administered for 6 weeks, followed by a four-week recovery period. Statistical analyses included normality testing (Shapiro–Wilk), multiple t-tests or Mann–Whitney tests with false discovery rate correction for between-group comparisons, and two-way repeated measures ANOVA or mixed-effects models for longitudinal analysis. Both protocols induced lymphopenia without systemic toxicity but exhibited distinct immunological profiles. Protocol I promoted rapid and reversible lymphocyte suppression, whereas Protocol II induced a slower onset with sustained inhibitory signaling. Phenotypic analysis revealed dynamic shifts within lymphocyte populations, including a decline in the CD4/CD8 ratio that did not reach statistical significance. CD21^+^ lymphocytes were differentially affected: Protocol I maintained higher levels after an initial transient decrease, while Protocol II showed a progressive reduction with significant differences at multiple time points. These findings highlight that co-stimulation blockade combined with calcineurin inhibition enforces deeper functional suppression, whereas MMF/tacrolimus-based therapy allows partial recovery of immune compartments. This direct comparative analysis provides a critical framework for designing targeted immunosuppressive strategies for translational models.

## 1 Introduction

Large animal models play a pivotal role in bridging the gap between basic research and clinical application in translational medicine. Among them, the porcine model (*Sus scrofa*) has gained widespread acceptance due to its anatomical, physiological, and immunological similarities to humans (1). These features are particularly advantageous in cardiovascular research, where swine are frequently used to model myocardial infarction, heart failure, and other cardiac pathologies with high translational relevance (2).

In recent years, the use of porcine models has expanded to include studies involving cell therapy and transplantation of tissue-engineered constructs. These approaches often require the introduction of allogeneic or xenogeneic cells or tissues, which in turn necessitates the use of immunosuppressive regimens to prevent immune-mediated rejection (3, 4).

As highlighted by Enosawa and Kobayashi (5), one widely used strategy combines mycophenolate mofetil (MMF) with tacrolimus. MMF, a semisynthetic morpholinoethyl ester of mycophenolic acid, inhibits lymphocyte proliferation by selectively targeting the *de novo* purine synthesis pathway. In swine, it is typically administered oral at 20 mg/kg twice daily and has been used in combination with other immunosuppressants as a maintenance regimen. This protocol has achieved notable long-term xenograft survival, with some cardiac xenotransplants lasting up to 945 days (6). Despite these promising outcomes, the pharmacodynamic profile of MMF and tacrolimus in swine remains insufficiently characterized, and their long-term immunological effects are still under investigation.

In contrast to these conventional antiproliferative/calcineurin inhibitor combinations, more targeted strategies involving co-stimulation blockade have emerged. The use of immune checkpoint inhibitors such as abatacept offers a more targeted approach to immunosuppression. Abatacept is a fusion protein composed of the extracellular domain of cytotoxic T-lymphocyte-associated protein 4 (CTLA4) linked to an IgG1 Fc fragment, which works by selectively modulating T-cell activation through blockade of the CD80/CD86: CD28 costimulatory pathway. This mechanism inhibits the second signal required for full T-cell activation, thereby reducing T-cell–mediated immune responses while potentially minimizing systemic toxicity. Recent preclinical studies have demonstrated the feasibility of using CTLA4-Ig in genetically modified pigs, where the expression of porcine CTLA4-Ig (pCTLA4-Ig) has been associated with prolonged graft survival and reduced immune activation, supporting its potential as a novel immunosuppressive strategy in large-animal models of xenotransplantation (7). In a study conducted in pigs to evaluate whether human embryonic stem cell-derived cardiomyocytes could regenerate infarcted myocardium, animals were pharmacologically immunosuppressed using a combination of cyclosporine A, methylprednisolone, and abatacept (8).

Despite the increasing use of swine in immunologically relevant research, few studies have systematically and directly compared the immunological and clinical effects of different immunosuppressive strategies in this species. As a result, there is a pressing need for evidence-based, comparative data on protocols that are both effective and safe for reliable implementation in translational research settings.

Therefore, this pilot study provides a direct, longitudinal comparative analysis of mechanistically distinct immunosuppressive protocols in swine, evaluating not only clinical safety but also detailed changes in key immune subsets. Our objective was to identify on a preliminary basis a protocol that provides robust immune modulation with minimal adverse effects, thereby generating a critical evidence base for future studies involving cell-based therapies.

## 2 Materials and methods

### 2.1 Study design

All experimental procedures were conducted in accordance with the ethical standards set by the Ethics Committee on Animal Experiments of the Jesús Usón Minimally Invasive Surgery Centre (JUMISC), following EU Directive 2010/63/EU and local legislation from the Junta de Extremadura on the protection of animals used for scientific purposes (EXP-20200519). Animals were housed in an accredited facility at JUMISC under standard husbandry conditions. Euthanasia was performed under deep anesthesia via intravenous administration of potassium chloride (KCl, 2 mmol/kg) following the above mentioned legislation.

The study included eight Large White pigs (4 males and 4 females), with an average body weight of 33.9 ± 3.09 kg. All animals were randomly assigned to either one of the two immunosuppressive treatment groups (*n* = 4 per group; 2 males and 2 females per group). Group allocation was performed prior to treatment initiation, and all animals were housed under identical conditions throughout the study period.

Protocol I consisted of a single intravenous induction dose of Mycophenolate Mofetil (Roche Registration GmbH, Germany; MMF, 1 g) and Methylprednisolone (500 mg; Pfizer Manufacturing Belgium NV, Belgium) administered at the beginning of the study. This was followed by a maintenance regimen consisting of oral administration of MMF (50 mg) and Tacrolimus (10 mg; STADA Arznei mittel AG, Germany), given every 12 h.

Protocol II began with an induction phase comprising intravenous administration of Abatacept (12.5 mg/kg; CATALENT ANGNI S. R. L., Italy) and Methylprednisolone (500 mg). To reinforce the immunosuppressive effect, two booster doses of Abatacept (12.5 mg/kg) were administered intravenously in weeks 2 and 4. The maintenance treatment for this group involves daily oral administration of Cyclosporine A (10 mg/kg; TEVA Pharmaceutical Works Private Limited Company, Hungary) every 24 h.

Both immunosuppressive regimens were maintained until week 6. After this time point, treatment was discontinued, and animals continued to be monitored until the end of the study at week 10, allowing for the assessment of immune recovery and rebound effects following drug withdrawal.

**Figure 1:**
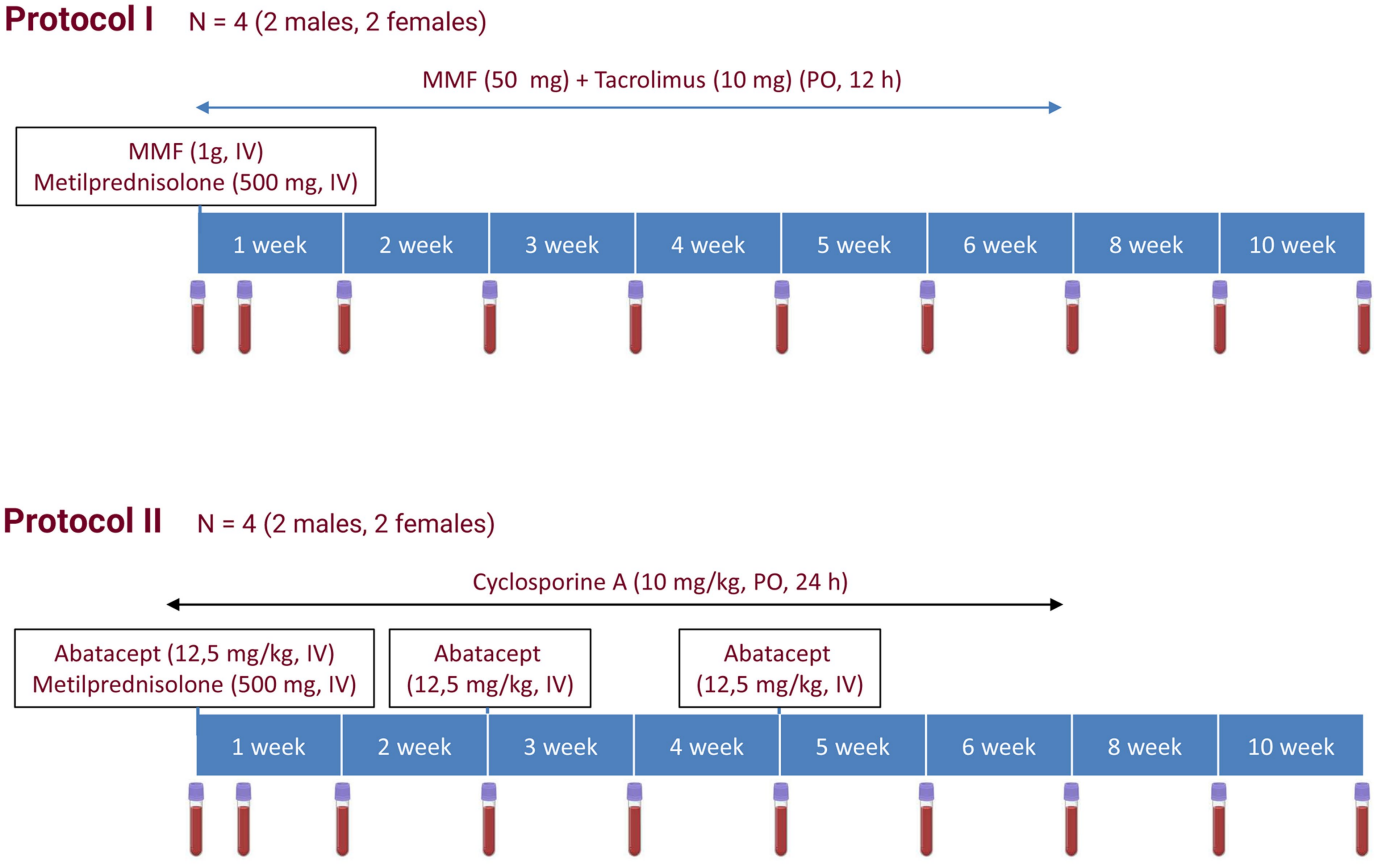
Workflow of materials and methods. The timeline illustrates the two immunosuppressive regimens used in the study. Both treatment options were administered for 6 weeks, followed by a 4-weeks drug-free monitoring period to evaluate immune recovery and potential rebound effects. MMF, Mycophenolate mofetil. IV, Intravenous administration. PO, Oral administration.

The study design is shown in Figure 1.

### 2.2 Blood collection

Approximately 16 mL of venous blood was collected from each pig into ethylenediaminetetraacetic acid (EDTA)-3 K tubes (BD Biosciences) at the following time points: day 0 (pre-treatment), day 2, and weeks 1, 2, 3, 4, 5, 6, 8, and 10 after initiation of the immunosuppressive regimen. The final two time points correspond to 2- and 4-week post-treatment cessation.

Whole blood was used for hematological analyses using an automatic hematology analyzer (Mindray BC-5300 Vet, Hamburg, Germany). Plasma was separated by centrifugation at 1200 × *g* for 5 min. Biochemical parameters were determined in the clinical analyzer Metrolab 2,300 (Metrolab S. A., Buenos Aires, Argentina) and the rest of plasma was stored at −80 °C.

Peripheral blood mononuclear cells (PBMCs) were isolated using Ficoll–Hypaque density gradient centrifugation (GE Healthcare, Illinois, United States) at 1200 × *g* for 20 min. Isolated PBMCs were washed twice with PBS and cryopreserved in liquid nitrogen for further analysis.

### 2.3 ELISA

To assess systemic immune activation status, plasma concentrations of interleukin-2 (IL-2) were measured using commercially available enzyme-linked immunosorbent assay (ELISA) kit (R&D Systems, MN, United States). All assays were performed according to the manufacturer’s protocols. Each sample was analyzed in duplicate.

### 2.4 Flow cytometry analysis

PBMCs were thawed and stained with the following fluorochrome-conjugated monoclonal antibodies: CD45 Pacific Blue (Bio-rad, CA, United States), CD4 PerCP (BD, New Jersey, United States), CD8*α* PE (BD, New Jersey, United States), CD45RA FITC (Bio-rad, CA, United States), CD279 CoraLite 647 (Proteintech, Illinois, United States), CD16 PE (Bio-rad, CA, United States), and CD21 PE (BD, New Jersey, United States). A total of 5 × 10^5^ cells were incubated with antibodies at 4 °C for 30 min, washed, and resuspended in PBS.

Samples were acquired on a MACSQuant 16 flow cytometer (Miltenyi Biotec), collecting data for 10^5^ events. Single-cell events were first identified by FSC-H versus FSC-A discrimination, followed by leukocyte gating based on CD45 expression. Within the CD45^+^ leukocyte population, CD4^+^ and CD8α^+^ lymphocytes were identified and subsequently characterized by CD45RA and CD279 (PD-1) expression to assess relative phenotypic states associated with differentiation and activation/exhaustion.

Within the same CD45^+^ leukocyte gate, CD21^+^ lymphocytes were used to identify B-cell–enriched populations, and CD16^+^ lymphocytes were used to define NK-cell-like populations, after excluding monocytes via FSC/SSC gating.

Data were analyzed using FlowLogic software (Agilent Technologies, Santa Clara, United States), with isotype-matched controls included in all experiments.

### 2.5 CD8α^+^ and CD4^+^ lymphocytes activation and intracellular IFN-*γ* detection

Cryopreserved PBMCs were seeded at 2 × 10^5^ cells/ml in 24-well plates and stimulated with a commercial T cell activation cocktail containing phorbol 12-myristate 13-acetate (PMA) and ionomycin (Invitrogen, Thermo Fisher, MA, United States). Brefeldin A (Invitrogen, Thermo Fisher, MA, United States) was added during stimulation to block cytokine secretion and allow intracellular accumulation.

After 24 h, cells were fixed, permeabilized, and stained with CD8α-PE (BD, New Jersey, United States) and IFN-*γ*-APC (BD, New Jersey, United States) monoclonal antibodies. Non-stimulated PBMCs served as negative controls. Samples were acquired on a MACSQuant 16 flow cytometer and analyzed using FlowLogic software.

### 2.6 Statistical analysis

All statistical analyses were performed using GraphPad Prism (version 9.5.1; GraphPad Software Inc.; San Diego, CA, United States). Data were first assessed for normality using the Shapiro–Wilk test. To compare differences between the two immunosuppression protocols at each time point, a multiple t-test approach was used. If the data followed a normal distribution, a parametric t-test was applied under no assumption about consistence SDs (Welch t-test) test. If normality was not met, a non-parametric Mann–Whitney U test was used instead.

In both cases, correction for multiple comparisons was applied using the False Discovery Rate (FDR) approach with the two-stage step-up method of Benjamini, Krieger and Yekutieli.

To assess changes over time within each protocol, a two-way repeated measures of ANOVA (or mixed-effects model in case of missing values) was performed. The Geisser–Greenhouse correction was applied to account for violations of sphericity. For post-hoc comparisons, each time point was compared to the corresponding baseline value (Day 0) within the same protocol, using Dunnett’s correction.

A significance threshold of *p* < 0.05 was used throughout all analyses.

## 3 Results

### 3.1 Animal monitoring and clinical observations

**Figure 2:**
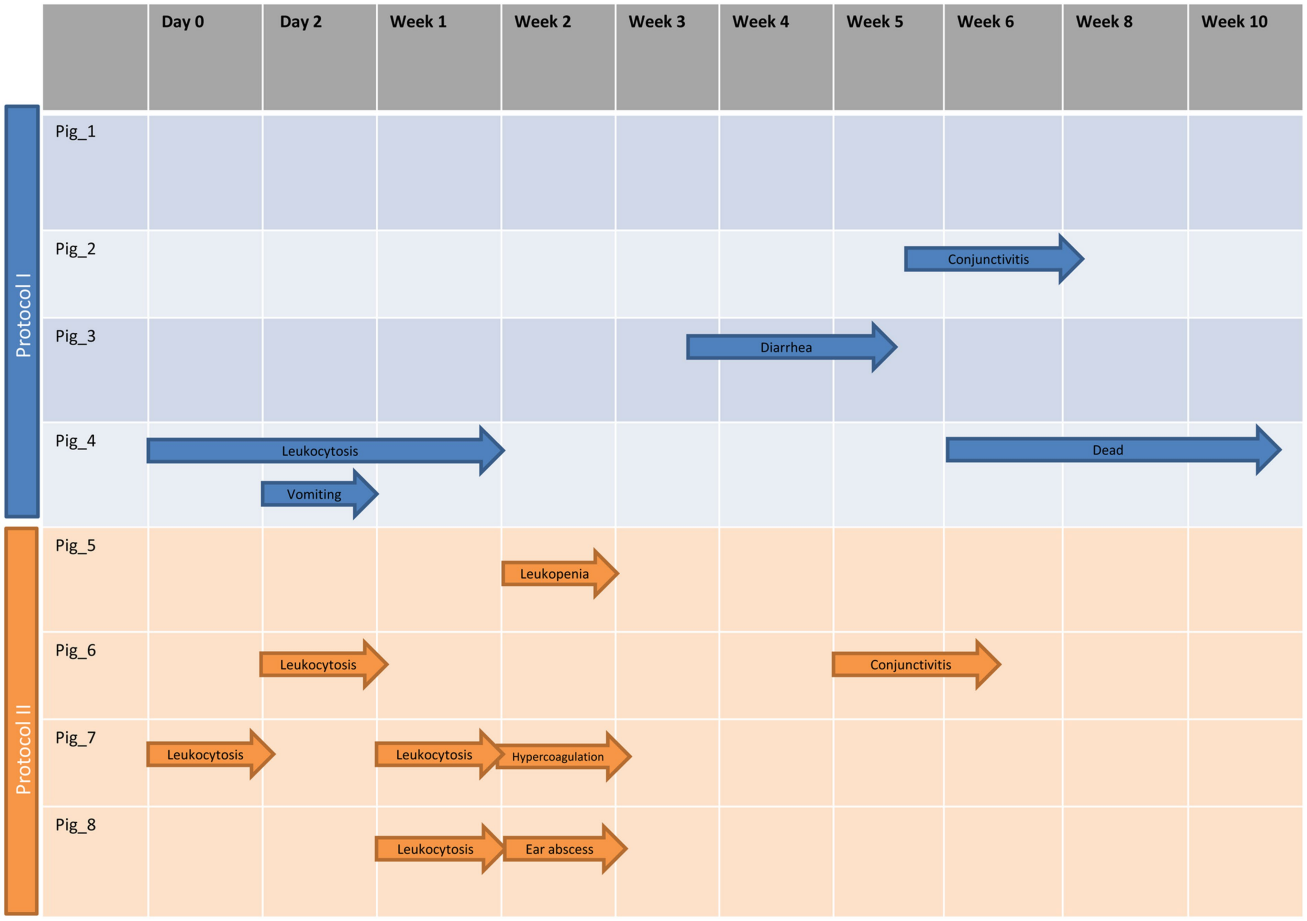
Adverse effects during the treatment timeline. A summary table of adverse effects identified during the treatment period, presented along the timeline. Animals treated with Protocol I are shown in blue, and those treated with Protocol II are shown in orange.

Throughout the study period, multiple clinical signs were observed among the animals, including diarrhea, vomiting, conjunctivitis, leukocytosis, and isolated cases of hypercoagulability and local abscess formation. Diarrhea occurred in several animals shortly after treatment initiation but resolved spontaneously in most cases. One animal experienced prolonged diarrhea accompanied by reduced appetite. Another animal died in week 5, likely due to general anesthesia, and was excluded from subsequent analyses. Conjunctivitis was noted in two animals. Despite these events, no consistent association was identified between the clinical signs and any specific immunosuppressive protocol (Figure 2).

### 3.2 Hematological and biochemical profiles

**Figure 3:**
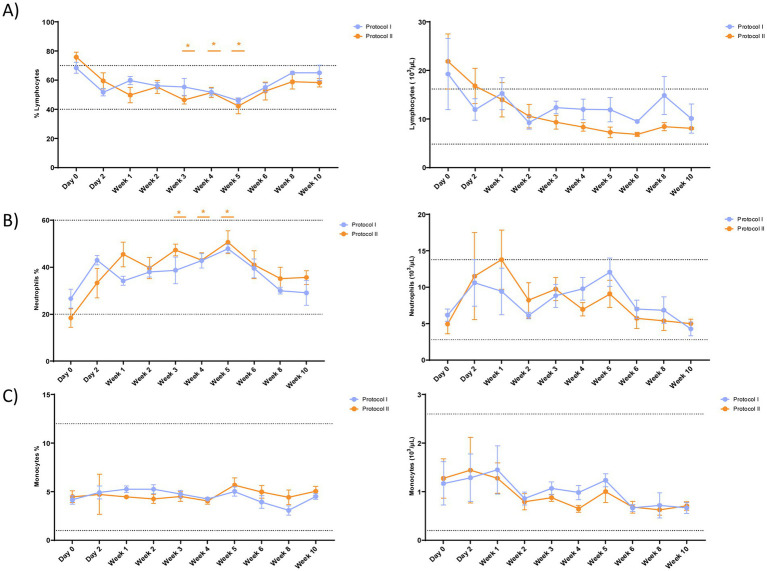
Effect of immunosuppressive protocols on hematological and biochemical parameters. **(A)** Lymphocyte percentage (top) and absolute lymphocyte count (bottom) over time. **(B)** Neutrophil percentage (top) and absolute neutrophil count (bottom). **(C)** Monocytes percentage (top) and absolute monocytes count (bottom). In all panels, blue represents Protocol I, and orange represents Protocol II. Data are presented as mean ± SD. The *p* values ≤0.05 were considered statistically significant. In all cases: *p < 0.05, **p < 0.005. Significance in Protocol I is highlighted in blue, and in Protocol II it is highlighted in orange. *p*-value corresponds to a mixed-effect analysis. Within-protocol statistical comparisons were performed relative to baseline (Day 0).

A hematological analysis was performed to assess the immunosuppressive efficacy and safety of two treatment protocols, with a primary focus on lymphocytes as a surrogate marker of immune suppression. Both regimens effectively reduced lymphocyte populations, confirming their immunomodulatory effects. The percentage of lymphocytes followed a similar trend in both protocols, with a marked decrease from the beginning of treatment and a subsequent increase after treatment withdrawal. However, significant differences were observed only in protocol II–treated animals, with values decreasing with values decreasing from day 0 (75.83%) to week 3 (46.48%; *p* = 0.0399), week 4 (51.58%; *p* = 0.0254), and week 5 (42.28%; *p* = 0.0399; Figure 3A). Although no significant differences were found in absolute lymphocyte counts, a reduction was observed in both protocols, being more pronounced in pigs submitted to protocol II (Figure 3A).

In contrast to lymphocytes, the percentage of neutrophils showed an increasing trend in both protocols. However, significant differences were observed only in protocol II–treated animals, with values increasing from day 0 (18.35%) to week 3 (47.28%; *p* = 0.0368), week 4 (42.88%; *p* = 0.0153), and week 5 (50.65%; *p* = 0.0300; Figure 3B). Absolute neutrophil counts, as well as their percentages, increased initially and subsequently declined from week 5 onward in both protocols; however, no statistically significant differences were detected (Figure 3B).

Regarding monocytes, the percentage remained relatively unchanged, but the absolute monocyte count decreased in both protocols starting from week 1, without reaching statistical significance (Figure 3C).

No significant differences were found between protocols in any of the leukocyte subsets.

Biochemical parameters showed no signs of hepatic or renal toxicity, with liver enzyme levels remaining within normal physiological ranges throughout the study. In addition, white blood cells, red blood cells, hemoglobin, and platelet counts were also maintained within their respective physiological ranges, further supporting the overall safety of the treatment regimen (Supplementary Figure 1).

### 3.3 Serum IL-2 concentration

**Figure 4:**
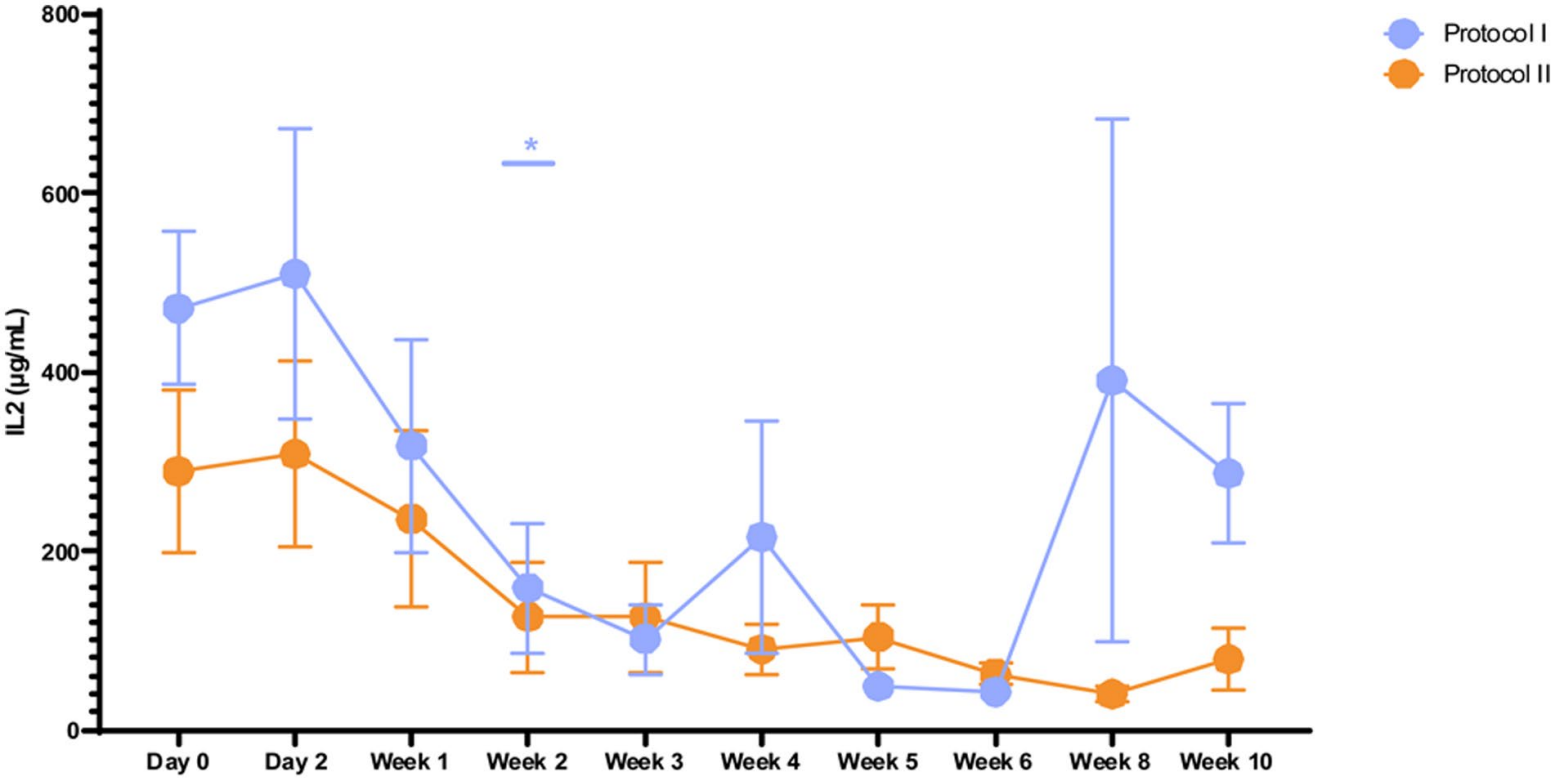
Effect of immunosuppressive protocols on IL-2 levels. Concentration of IL-2 is measured in μg/ml. Blue represents Protocol I and orange represents Protocol II. Data are presented as mean ± SD. The *p* values ≤ 0.05 were considered statistically significant. In all cases: *p < 0.05, **p < 0.005 Significance in Protocol I is highlighted in blue, and in Protocol II it is highlighted in orange. *p*-value corresponds to a mixed-effect analysis. Within protocol statistical comparisons were performed relative to baseline (Day 0).

To evaluate the immunosuppressive impact of the treatments, IL-2 serum concentrations were measured. IL-2, a cytokine associated with T cell activation and proliferation, served as an indicator of immune modulation. Both protocols induced a progressive decline in IL-2 concentrations during the immunosuppressive phase (Figure 4), consistent with effective suppression of T cell activation. Following withdrawal of treatment at week 6, IL-2 levels in protocol I–treated pigs increased from week 6 (43 μg/mL; *p* = 0.1854) to week 8 (390.24 μg/mL; *p* = 0.9997), and then decreased at week 10 (286.04 μg/mL; *p* = 0.7253). In protocol II–treated pigs, levels remained relatively stable, ranging from week 6 (62.94 μg/mL; *p* = 0.2438) to week 8 (40.12 μg/mL; *p* = 0.2758) and week 10 (79.17 μg/mL; *p* = 0.3661). Although these changes were not statistically significant.

### 3.4 Expression of CD45RA and CD279 in CD4^+^ and CD8*α*^+^ lymphocytes

**Figure 5:**
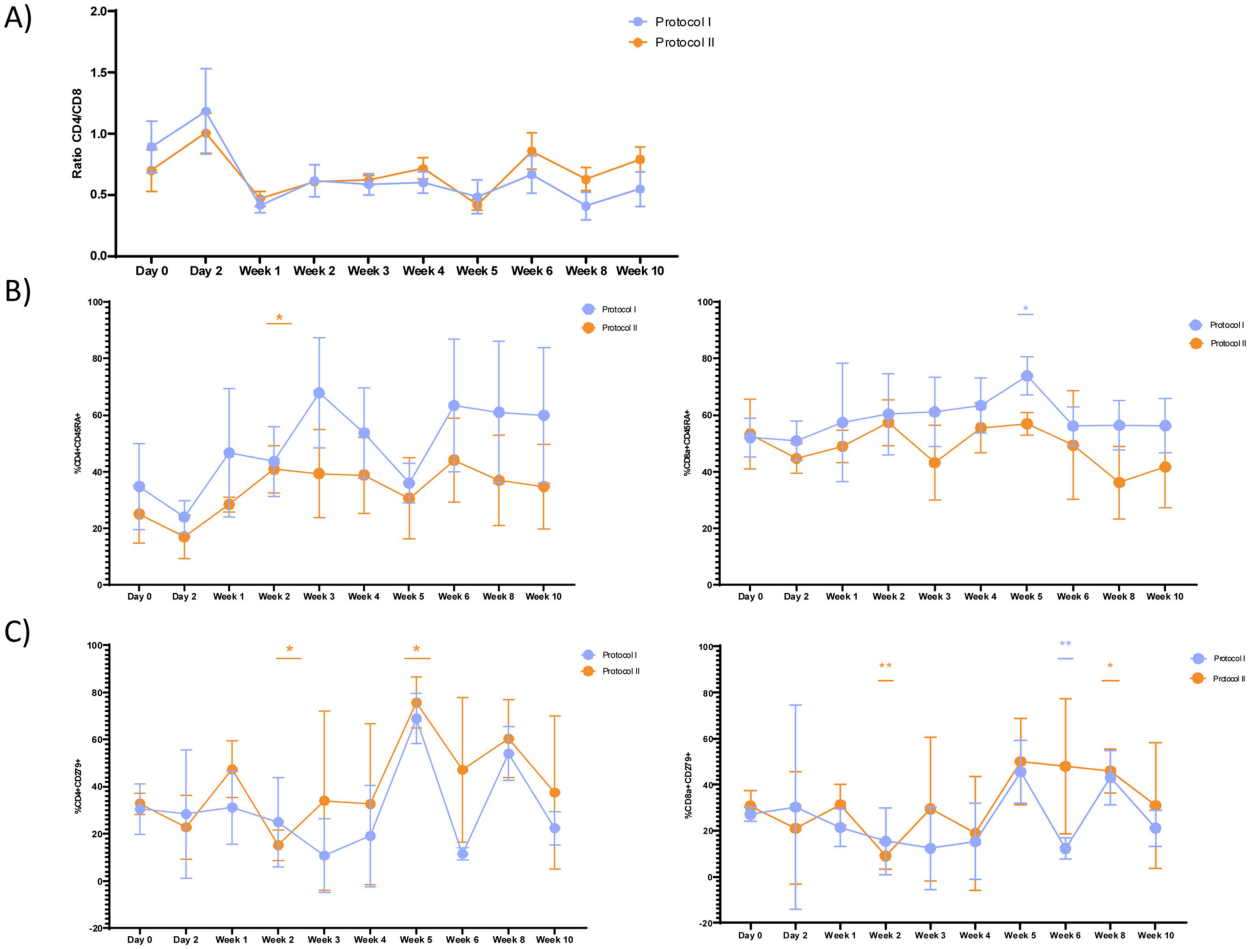
Effect of immunosuppressive protocols on CD4 and CD8α lymphocytes. **(A)** CD4/CD8 ratio **(B)** Percentage of CD45RA^+^ lymphocytes **(C)** Percentage of CD279^+^ lymphocytes. In all panels, blue represents Protocol I, and orange represents Protocol II. Data are presented as mean ± SD. The *p* values ≤ 0.05 were considered statistically significant. In all cases: *p < 0.05, **p < 0.005. Significance in Protocol I is highlighted in blue, and in Protocol II it is highlighted in orange. *p*-value corresponds to a mixed-effect analysis. Within-protocol statistical comparisons were performed relative to baseline (Day 0).

To further characterize the CD4^+^ and CD8α^+^ cell subsets under the regimens, flow cytometric analysis was performed on peripheral blood mononuclear cells under non-activated conditions. First, the analysis of the CD4/CD8 ratio revealed a similar decreasing trend in both treatments starting from day 2 (Figure 5A).

Assessment of absolute cell counts showed a decrease in both CD4^+^ and CD8*α*^+^ lymphocyte numbers over time. A statistically significant reduction in CD8*α*^+^ cell counts was observed under Protocol II, whereas changes in absolute CD4^+^ cell numbers did not reach statistical significance. Despite the decrease in absolute CD4^+^ counts, the relative proportion of CD4^+^ lymphocytes remained largely stable, while the proportion of CD8*α*^+^ lymphocytes increased (Supplementary Figure 3).

Regarding phenotypic profiles, CD45RA expression increased in both CD4^+^ and CD8*α*^+^ subsets across protocols, but with notable differences. In the CD4^+^ population, both protocols showed an early rise in CD45RA^+^ frequency from baseline, becoming significantly only in Protocol II at week 2 (*p* = 0.049). For the CD8α^+^ subset, both protocols displayed similar levels at baseline, but Protocol I exhibited a progressive increase reaching a significant difference at week 5 (*p* = 0.047; Figure 5B).

Conversely, CD279 (PD-1) expression exhibited distinct dynamics between protocols. In the CD4^+^ subset, Protocol I showed an initial reduction until week 3, followed by a pronounced peak at week 5, a decrease at week 6, and a subsequent increase later in the study. In contrast, Protocol II maintained relatively stable levels, but after reaching its lowest point at week 2 (from 32.8% at baseline to 15.22%, *p* = 0.033), levels began to rise, becoming significantly higher by week 5 (75.65%, *p* = 0.017). In the CD8α^+^ subset, both protocols displayed a comparable pattern characterized by an early reduction (more pronounced in Protocol II) followed by a progressive increase from mid-treatment onward. A marked increase in CD279 expression was observed in Protocol II after week 4, while Protocol I exhibited transient peaks before declining again at week 6 (Figure 5C).

### 3.5 CD4^+^ and CD8*α*^+^ lymphocytes activation

To assess the functional capacity of CD4^+^ and CD8*α*^+^ lymphocytes under immunosuppressive conditions, we measured the production of interferon-gamma (IFN-*γ*) by CD4^+^ and CD8α^+^ lymphocytes following ex vivo PBMC stimulation with PMA and ionomycin.

**Figure 6:**
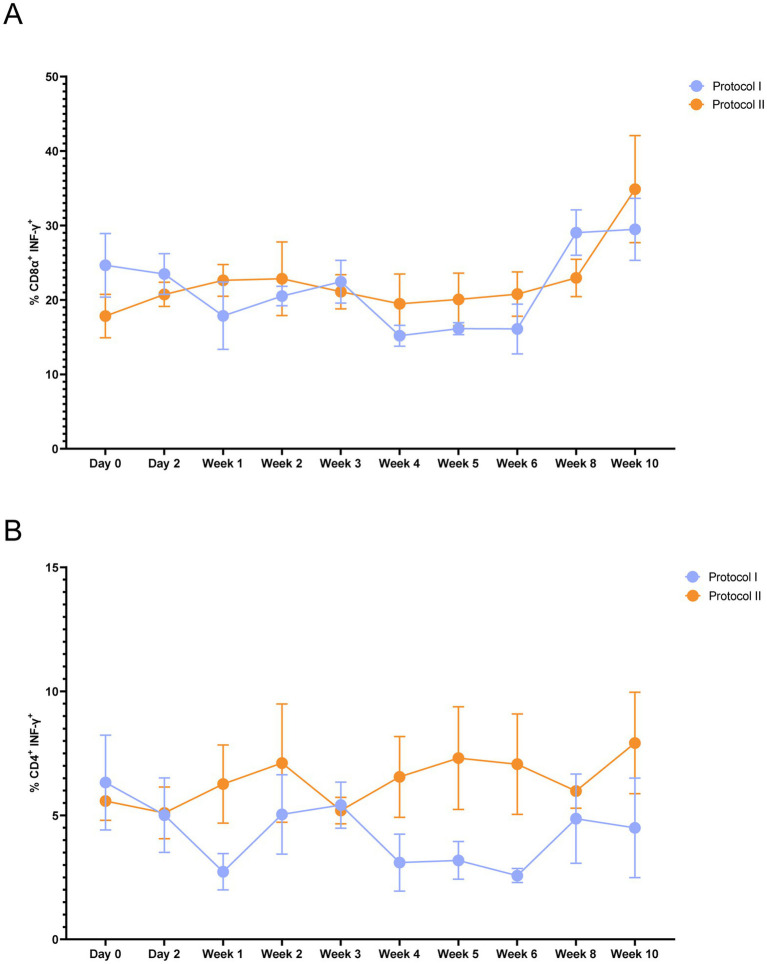
Effect of immunosuppressive protocols on IFN-*γ* secretion by CD8α^+^ and CD4^+^ lymphocytes following activation. **(A)** Percentage of CD8α^+^IFN-γ^+^ lymphocytes. **(B)** Percentage of CD4^+^IFN-γ^+^ lymphocytes. In all panels, blue represents Protocol I, and orange represents Protocol II. Data are presented as mean ± SD. Statistical significance was determined using a threshold of *p* ≤ 0.05. Significance levels are indicated as follows: *p < 0.05, **p < 0.005. Significance in P rotocol I is highlighted in blue, and in Protocol II it is highlighted in orange. *p*-value corresponds to a mixed-effect analysis. Within-protocol statistical comparisons were performed relative to baseline (Day 0).

In protocol I, a reduction in both the percentage of CD8*α*^+^ IFN-γ^+^ lymphocytes and the percentage of CD4^+^ IFN-γ^+^ was observed as early as week 1, and this suppression persisted throughout the treatment period up to week 6. Notably, following treatment withdrawal, a sharp rebound in IFN-γ production was detected at weeks 8 and 10 in both CD4^+^ and CD8*α*^+^ lymphocytes, with levels exceeding baseline values only in the case of CD8α^+^ lymphocytes (Figure 6A).

In contrast, IFN-γ production by CD8*α*^+^ and CD4^+^ lymphocytes from animals treated with Protocol II remained relatively stable during the treatment phase, with a progressive increase reaching a peak after week 6 (Figure 6B).

However, despite these apparent trends, no statistically significant differences at any time point were observed between time points or treatment groups.

### 3.6 CD21^+^ lymphocytes

**Figure 7:**
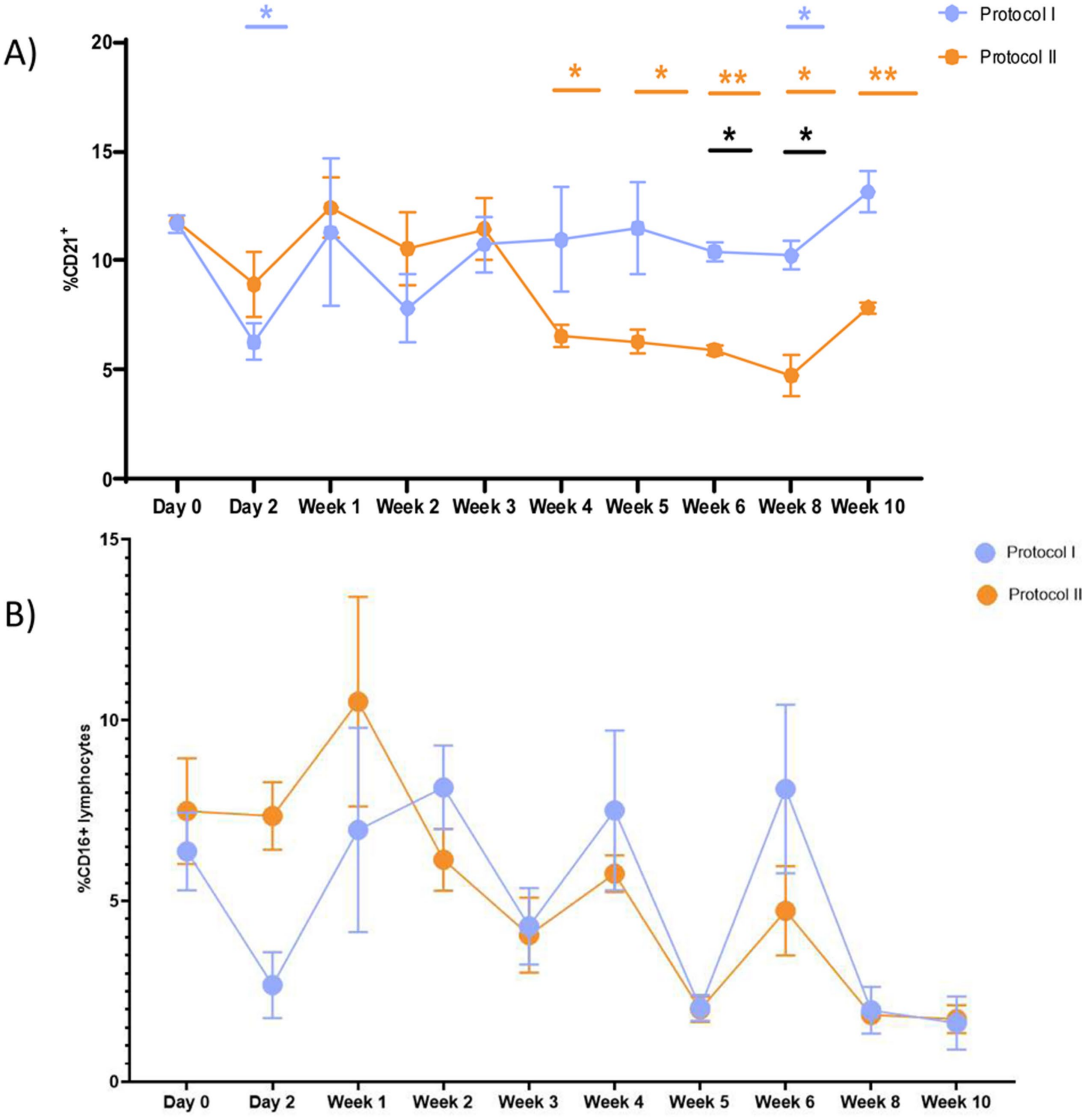
Effect of immunosuppressive protocols on CD21^+^ and CD16^+^ lymphocytes subsets. **(A)** Percentage of CD21^+^ lymphocytes over time in both treatment groups. **(B)** Percentage of CD16^+^ lymphocytes. In all panels, blue represents Protocol I, and orange represents Protocol II. Data are presented as mean ± SD. The *p* values ≤ 0.05 were considered statistically significant. In all cases: *p < 0.05, **p < 0.005. Significance in Protocol I is highlighted in blue, in Protocol II it is highlighted in orange, and between protocols it is highlighted in black. *p*-values correspond to mixed-effects analysis (within-group) or *t*-test (between groups). Within-protocol statistical comparisons were performed relative to baseline (Day 0).

The percentage of CD21^+^ lymphocytes was also influenced by immunosuppressive regimens. By Day 2, Protocol I showed a transient decrease followed by recovery, while Protocol II maintained relatively stable levels. From week 3 onwards, Protocol I consistently maintained higher expression of CD21^+^ lymphocytes. However, Protocol II decreased the percentage of CD21^+^ lymphocytes with statistically significant differences at weeks 4, 5, 6, 8, and 10 compared with their basal. Both protocols displayed a progressive decline after a slight recovery by week 10. Interestingly, the trend of both protocols was different with significant differences in week 6 and 8 (Figure 7A).

### 3.7 CD16^+^ lymphocytes

The percentage of CD16 + lymphocytes also declined in animals treated with Protocol II, beginning at week 2. In contrast, in animals treated with Protocol I, CD16^+^ lymphocytes levels remained relatively stable, showing intermittent fluctuations throughout the treatment period (Figure 7B).

## 4 Discussion

Cell-based therapies, including cell replacement and tissue transplantation strategies, rely on immunosuppressive regimens to prevent graft rejection and ensure therapeutic efficacy. This is particularly relevant in regenerative medicine, where the successful survival of transplanted cells requires temporary modulation of the host immune system. However, prolonged or excessive immunosuppression can severely compromise immune competence, increasing the risk of opportunistic infections, tumors, and impaired tissue remodeling (9, 10). Thus, the development of potent and reversible immunomodulatory remains a central challenge in advancing toward clinical translation.

In this context, the pig has emerged as a valuable large-animal model for preclinical research, owing to its anatomical, physiological, and immunological similarities to humans. Its suitability for surgical and interventional procedures, combined with its translational relevance, makes it an ideal platform for evaluating the safety and efficacy of immunosuppressive protocols under conditions that closely mimic human physiology. Establishing immunosuppressive regimens that effectively modulate both innate and adaptive immune compartments, without inducing systemic toxicity, are essential for xenogeneic cell-based medicinal products.

Here we directly evaluated and compared two immunosuppressive regimens (protocol I and protocol II). Both regimens were well tolerated, with no evidence of toxicity as demonstrated by hematological and biochemical parameters. It is important to note that regimens based on calcineurin inhibitors, such as tacrolimus and cyclosporine A, alone or in combination with other immunosuppressants, have long been established as cornerstone agents in transplant immunosuppression (11, 12). Moreover, their effects are reversible, and upon withdrawal of treatment T cell activation and proliferation are restored (13).

The observed recovery in the percentage of peripheral blood lymphocytes following withdrawal of treatments (protocol I and protocol II) aligns with previous findings indicating that calcineurin inhibition leads to transient immunosuppression, allowing for partial immune reconstitution once the drug is cleared (14, 15). This feature is particularly advantageous in preclinical studies involving xenogeneic cell-based models where temporary immune modulation is sufficient to permit survival without compromising long-term immunity.

On the other hand, corticosteroids exert broader immunosuppressive effects, including the mobilization of leukocytes, inhibition of antigen-presenting cell function, and suppression of pro-inflammatory cytokine production (16). In our experimental conditions, the neutrophilia observed under both protocols agrees with corticosteroid-induced neutrophil mobilization and enhanced neutrophil survival (17, 18).

The novelty of this study lies in the systematic, longitudinal comparison of a conventional antiproliferative/calcineurin-inhibitor regimen (MMF/Tacrolimus) versus a targeted co-stimulation blockade/calcineurin-inhibitor regimen (Abatacept/Cyclosporine) in a translational porcine model. While both protocols have been used individually, their direct comparative immunological profiles have not been described. Our first-level hematological analysis confirmed core immunosuppression without toxicity in both groups. However, given their mechanistic differences, we performed a deeper comparative evaluation of lymphocyte subsets via flow cytometry to uncover distinct immunological footprints.

In our phenotypic analysis, we first determined the CD4/CD8 ratio, which is a well-established marker of immune system activation and dysregulation. In both protocols, we observed a non-significant reduction of the ratio during the first weeks of treatment. When absolute cell counts were considered, both CD4^+^ and CD8α^+^ lymphocyte numbers declined over time, with a statistically significant reduction of CD8α^+^ lymphocytes observed in Protocol II. However, despite the decrease in absolute CD4^+^ counts, the relative proportion of CD4^+^ lymphocytes remained stable, whereas the proportion of CD8α^+^ lymphocytes increased. Although we found that the decrease was not statistically significant, previous studies have found a significant reduction of CD4/CD8 ratio and intra-patient variability in kidney transplant recipients treated with tacrolimus (19). Similarly, a decrease of CD4/CD8 ratio has previously been associated with minimized alloreactivity and a reduced risk of rejection (20).

Phenotypic analysis of CD4^+^ and CD8α^+^ lymphocytes revealed that the two immunosuppressive protocols induce distinct immune profiles in this porcine model. Protocol I was associated with a higher proportion of CD45RA^+^ lymphocytes compared with Protocol II, suggesting differential modulation of the peripheral immune landscape under each regimen.

In transplantation settings, the composition and differentiation state of the recipient immune system are key determinants of graft acceptance and immune reactivity. Modulation of CD45RA^+^ populations has been associated with altered alloreactive potential, as depletion of CD45RA^+^ cells reduces naïve alloreactive T-cell content and improves transplantation outcomes (21). In contrast, CD279 (PD-1), a marker of activation, regulation, or exhaustion, displayed distinct kinetics. The transient modulation of PD-1 expression observed across protocols may indicate a shift from early cell activation to later regulatory control. It is known that PD-1 upregulation after allogenic cell therapies serves as a physiological mechanism to restrain donor T-cell activity and prevent excessive alloreactivity (22), while sustained PD-1 expression on CD4^+^ cells has been linked to regulatory or exhausted phenotypes that maintain peripheral tolerance (23). This later increase in our results of PD-1 expression could therefore favor an immunosuppressive milieu.

Cytokine profiling, particularly IL-2 and IFN-*γ*, offers a functional readout of T cell activity. IL-2 is a pivotal cytokine for T cell proliferation and survival, and its suppression is a hallmark of effective immunosuppression (24, 25). This is reflected in the reduced IL-2 levels achieved with both protocols, more pronounced under Protocol I. The differential recovery in IL-2 levels between protocols suggests distinct kinetics of immune recovery, with Protocol I promoting a more rapid restoration of T cell function. This pattern suggests a partial recovery of T cell function, which was not evident in Protocol II. Similarly, IFN-γ production by lymphocytes reflects effector capacity and immune surveillance potential. Although inter-animal variability limited statistical significance, the trends observed are consistent with prior reports. Protocol I induced early suppression in both stimulated CD8α^+^ and CD4^+^, followed by a marked rebound after treatment cessation (25, 26). Protocol II, on the other hand, showed a gradual increase over time, consistent with reports indicating that cyclosporine A, although also a calcineurin inhibitor, may exert immunostimulatory effects on CD8α^+^ lymphocytes (27).

CD21 ^+^ lymphocytes dynamics also reflect the immunosuppressive pressure exerted by each protocol. Among all immune subsets analyzed, changes in the percentage of CD21^+^ lymphocytes were particularly pronounced, underscoring their sensitivity to pharmacological modulation. The progressive decline, especially observed in Protocol II, suggests that the inclusion of abatacept in Protocol II may contribute to a deeper impact on B-cell homeostasis. Mechanistically, abatacept has been shown to directly modulate B cells by reducing CD80 and CD86 expression through a dynamin-dependent internalization process, ultimately lowering plasmablast frequencies and IgG production (28). These findings support the concept that immunosuppressive strategies do not act exclusively on T cells but orchestrate a broader remodeling of adaptive immunity (29).

Within the context of optimizing immunosuppressive regimens for cell therapy, monitoring B-cell subsets based on CD21 expression provides valuable insights into immune regulation. CD21^−^/low B cells largely represent activated or memory populations associated with chronic immune stimulation or dysregulation (30). Following allogeneic cell therapy, B-cell reconstitution typically involves an early expansion of CD21^−^/low transitional cells that gradually mature into CD21hi subsets (31). Consequently, an immunosuppressive regimen that preserves CD21^+^ populations while limiting excessive activation could foster a controlled immune environment, thereby minimizing innate B-cell reactivity and promoting graft tolerance.

In line with the immune modulation observed across our protocols, experimental models have long demonstrated that carefully balanced immunosuppressive regimens are essential to promote graft tolerance. These findings provide a mechanistic foundation for applying controlled immunosuppression to enhance the engraftment and persistence of cellular therapies. Although MSC-based therapies generally do not require immunosuppression due to their low immunogenicity (32), the allogeneic application of other cell therapies, such as iPSCs, still depends on immunosuppressive strategies because of their limited engraftment potential (33). Emerging approaches, such as the use of hypoimmune iPSCs engineered to evade immune detection, may eventually reduce or even eliminate the need for pharmacological immunosuppression (34). Additionally, co-transplantation with immunomodulatory cells, such as iPSC-derived mesenchymal stromal cells, has shown promise in inducing tolerance and enhancing graft survival without sustained immunosuppression (35).

In interpreting these findings, several limitations should be considered. First, the study was designed as an exploratory pilot experiment using a small number of large animals, which inherently limits statistical power and the ability to detect subtle immunological differences between treatment groups. However, given the ethical constraints associated with large animal experimentation this small cohort was selected as sufficient to set the basis for future studies requiring immune suppression in swine. Biological variability within the porcine model, together with the death of one animal at week 5, may also have influenced the interpretation and consistency of certain clinical and immunological parameters. Finally, the duration of follow up, while sufficient to evaluate treatment withdrawal and early immune recovery, may not capture long term or delayed immunological consequences.

The choice of induction therapy is a critical determinant of transplant outcomes, as both induction and maintenance phases contribute to the overall success of immunosuppressive regimens. In our study, Protocol I and Protocol II differ substantially in both induction and maintenance, with Protocol I using a single dose of MMF and methylprednisolone followed by a maintenance regimen of MMF and tacrolimus, and Protocol II employing abatacept with multiple booster doses and methylprednisolone followed by a maintenance regimen of cyclosporine A. The immunological differences observed between the protocols likely reflect the combined effects of induction and maintenance, and our design does not allow the separate evaluation of their individual contributions. Evidence from human transplantation and recent xenotransplantation studies demonstrates that induction strategies influence graft survival, regulatory T cell activation, immune exhaustion, and tolerance mechanisms (36–40). Importantly, this study does not include a group receiving only induction therapy without subsequent maintenance, so the specific contribution of induction versus maintenance cannot be determined. Although this pilot study provides a preliminary direct, evidence-based comparison that serves as a decision-making framework for selecting immunosuppressive regimens according to the therapeutic objective, the nature of the graft, and the desired balance between immune protection and immune restoration, these limitations should be taken into account when interpreting the findings. Even so, the study offers a solid foundation for future larger-scale and mechanistically focused investigations.

## Data Availability

The original contributions presented in the study are included in the article/Supplementary material, further inquiries can be directed to the corresponding author.

## Supplementary material

SUPPLEMENTARY FIGURE 1 **(A)** Hematological markers; **(B)** biochemical markers. GGT, Gamma-glutamyl transferase. GOT, glutamic oxaloacetic transaminase. GPT, glutamic pyruvic transaminase.

SUPPLEMENTARY FIGURE 2 Gating strategy. Single-cell events were first identified by discrimination of FSC-H versus FSC-A, followed by gating of leukocytes based on CD45 expression. Within the CD45^+^ leukocyte population, CD4^+^ and CD8α^+^ lymphocytes were identified and further characterized according to CD45RA and CD279 (PD-1) expression to evaluate phenotypic states associated with differentiation and activation/exhaustion. Within the same CD45^+^ leukocyte gate, CD21^+^ lymphocytes were used to define B cell–enriched populations, while CD16^+^ lymphocytes were used to identify NK-like populations after exclusion of monocytes based on FSC/SSC parameters.

SUPPLEMENTARY FIGURE 3 Absolute changes in CD4^+^ and CD8α^+^ lymphocyte populations over time. **(A)** Absolute counts of CD4^+^ lymphocytes. **(B)** Absolute count of CD8α^+^ lymphocytes. In all panels, blue represents Protocol I, and orange represents Protocol II. Data are presented as mean ± SD. The p values ≤ 0.05 were considered statistically significant. In all cases: **p* < 0.05, ***p* < 0.005. Significance in Protocol I is highlighted in blue, and in Protocol II it is highlighted in orange. *p*-value corresponds to a mixed-effect analysis. Within-protocol statistical comparisons were performed relative to baseline (Day 0).

SUPPLEMENTARY TABLE 1 Complete list of all antibodies used in the study, including clone, fluorochrome, provider, and catalog number.
