# Supplementary figures and images for: A pilot study investigating the comparison of immunological responses to two immunosuppressive regimens in a porcine model
Source: Front Vet Sci. 2026 Jun 17;13:1816404. doi: 10.3389/fvets.2026.1816404 (PMC13318597; doi:10.3389/fvets.2026.1816404)

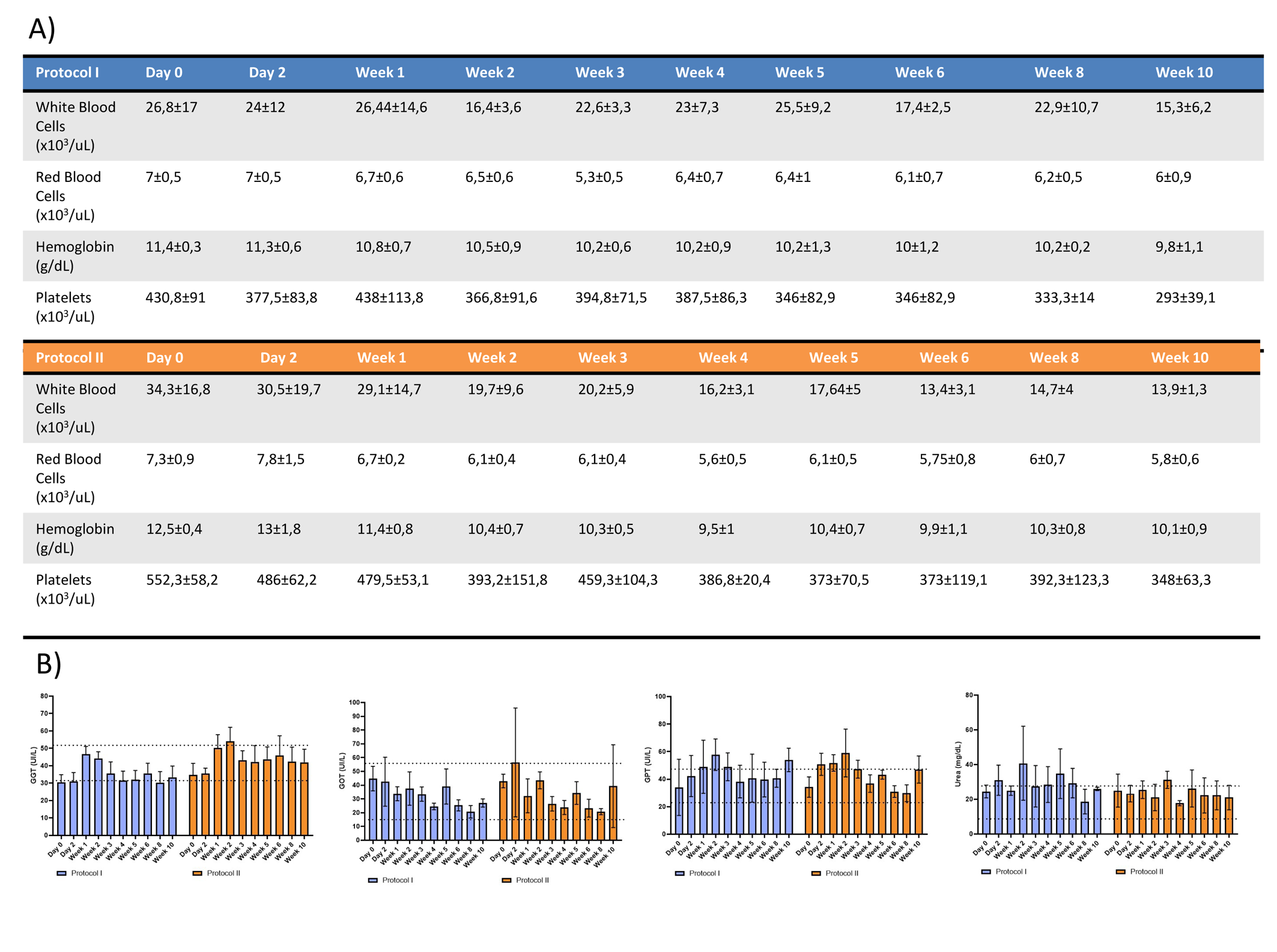

Supplement: SUPPLEMENTARY FIGURE 1 — (A) Hematological markers; (B) biochemical markers. GGT, Gamma-glutamyl transferase. GOT, glutamic oxaloacetic transaminase. GPT, glutamic pyruvic transaminase. [file Image_1.TIF]

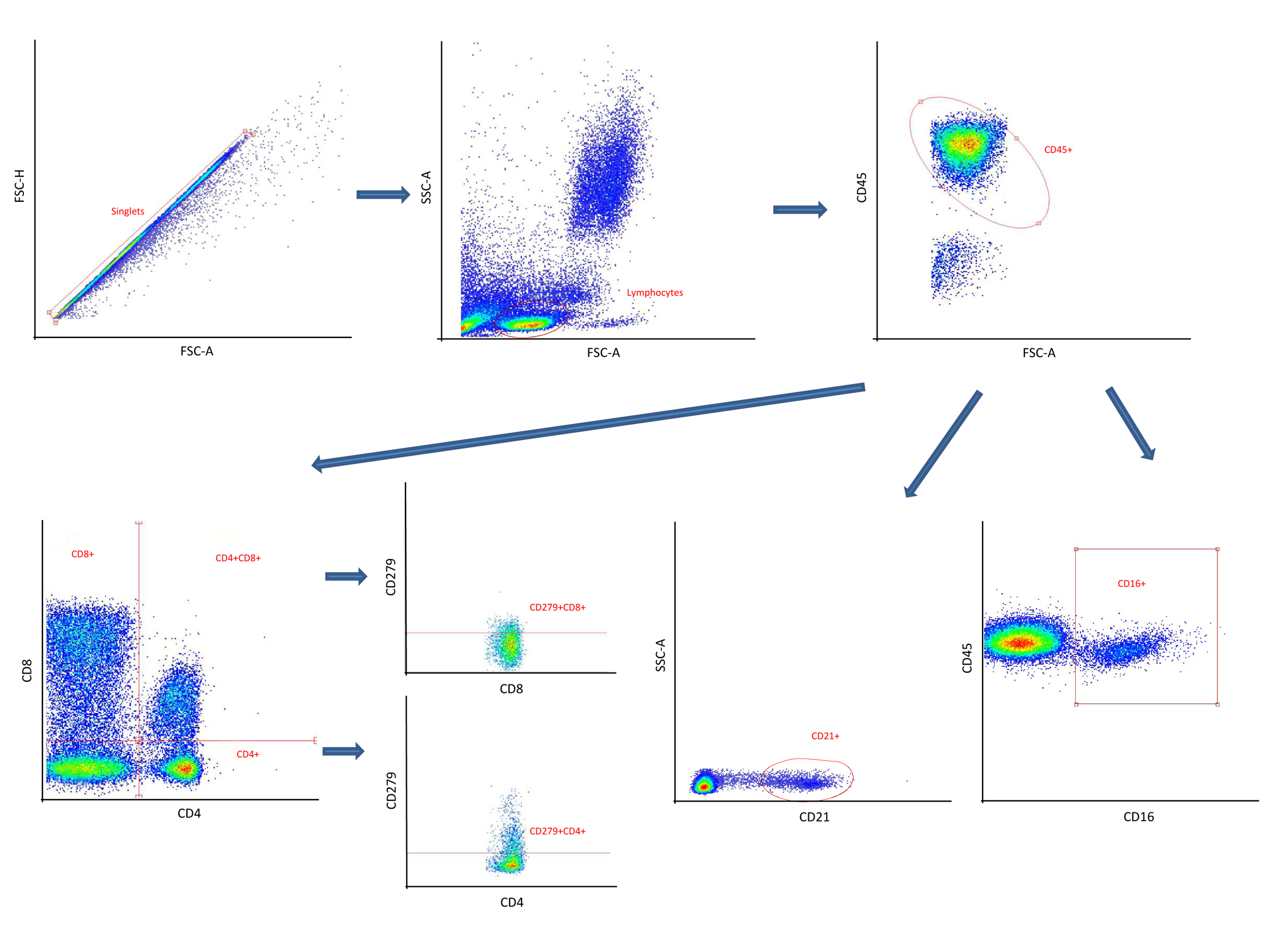

Supplement: SUPPLEMENTARY FIGURE 2 — Gating strategy. Single-cell events were first identified by discrimination of FSC-H versus FSC-A, followed by gating of leukocytes based on CD45 expression. Within the CD45+ leukocyte population, CD4+ and CD8α+ lymphocytes were identified and further characterized according to CD45RA and CD279 (PD-1) expression to evaluate phenotypic states associated with differentiation and activation/exhaustion. Within the same CD45+ leukocyte gate, CD21+ lymphocytes were used to define B cell–enriched populations, while CD16+ lymphocytes were used to identify NK-like populations after exclusion of monocytes based on FSC/SSC parameters. [file Image_2.TIF]

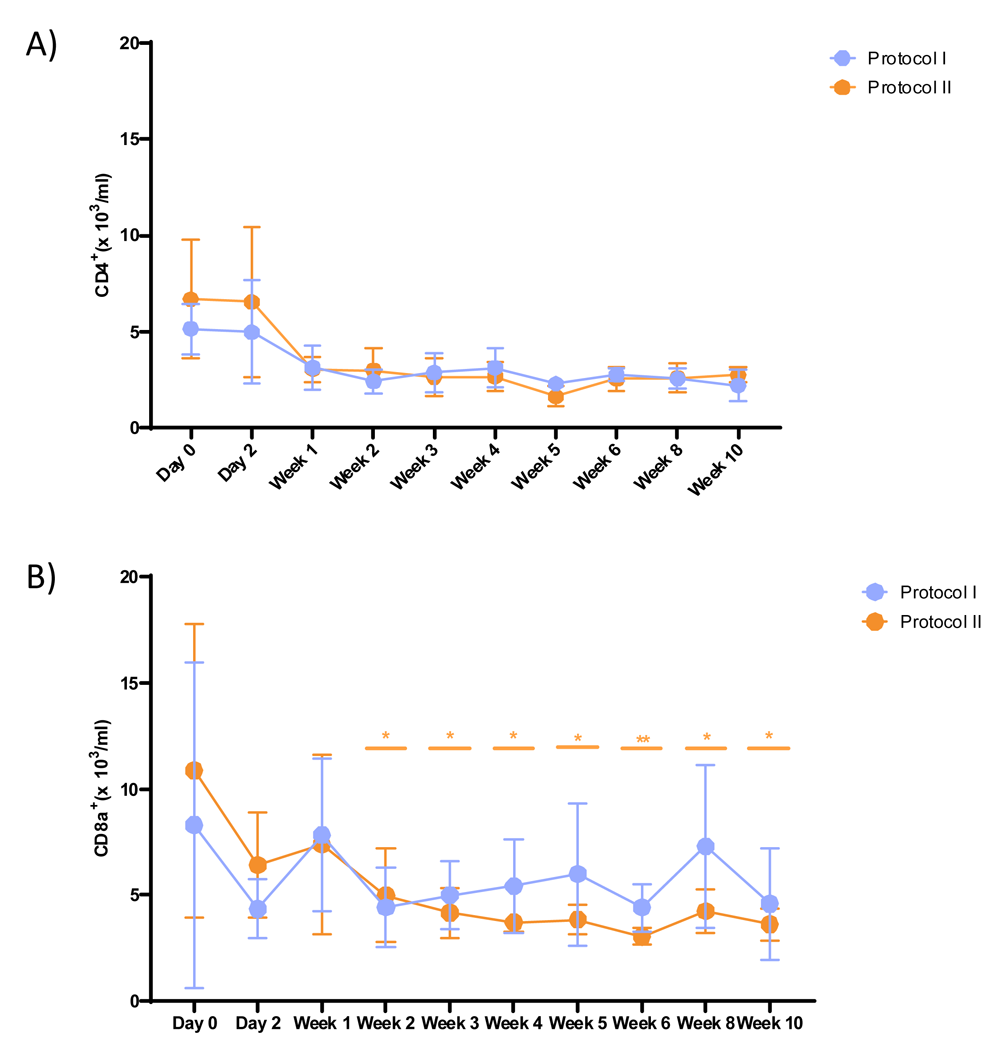

Supplement: SUPPLEMENTARY FIGURE 3 — Absolute changes in CD4+ and CD8α+ lymphocyte populations over time. (A) Absolute counts of CD4+ lymphocytes. (B) Absolute count of CD8α+ lymphocytes. In all panels, blue represents Protocol I, and orange represents Protocol II. Data are presented as mean ± SD. The p values ≤ 0.05 were considered statistically significant. In all cases: *p < 0.05, **p < 0.005. Significance in Protocol I is highlighted in blue, and in Protocol II it is highlighted in orange. p-value corresponds to a mixed-effect analysis. Within-protocol statistical comparisons were performed relative to baseline (Day 0). [file Image_3.TIF]
