# Supplementary material for: A pilot study investigating the comparison of immunological responses to two immunosuppressive regimens in a porcine model
Source: Front Vet Sci. 2026 Jun 17;13:1816404. doi: 10.3389/fvets.2026.1816404 (PMC13318597; doi:10.3389/fvets.2026.1816404)
Supplement: SUPPLEMENTARY TABLE 1 — Complete list of all antibodies used in the study, including clone, fluorochrome, provider, and catalog number. [file Table_1.DOCX]

| Antibody | Fluorochrome | Clone | Provider | Catalog number |
| --- | --- | --- | --- | --- |
| CD45 | Pacific Blue | K252.1E4 | Bio-rad | MCA1222PB |
| CD4 | PerCp Cy5.5 | 74-12-4 | BD | 561474 |
| CD8a | PE | MIL12 | BD | MCA1223PE |
| CD45RA | FITC | MIL13 | Bio-rad | MCA1751F |
| CD279 (PD-1) | CoraLite 647 | 4H4D1 | Proteintech | CL647-66220 |
| CD16 | PE | G7 | Bio-rad | MCA1971PE |
| CD21 | PE | B-ly4 | BD | 557327 |
